# Exposure to Organophosphate and Neonicotinoid Insecticides and Its Association with Steroid Hormones among Male Reproductive-Age Farmworkers in Northern Thailand

**DOI:** 10.3390/ijerph18115599

**Published:** 2021-05-24

**Authors:** Neeranuch Suwannarin, Tippawan Prapamontol, Tomohiko Isobe, Yukiko Nishihama, Yuki Hashimoto, Ampica Mangklabruks, Tawiwan Pantasri, Somporn Chantara, Warangkana Naksen, Shoji F. Nakayama

**Affiliations:** 1 Ph.D. Degree Program in Environmental Science, Environmental Science Research Center, Faculty of Science, Chiang Mai University, Chiang Mai 50200, Thailand; suwannarin.ns@gmail.com; 2 Research Institute for Health Sciences (RIHES), Chiang Mai University, Chiang Mai 50200, Thailand; 3 Health and Environmental Risk Division, National Institute for Environmental Studies, Tsukuba, Ibaraki 305-8506, Japan; isobe.tomohiko@nies.go.jp (T.I.); nishihama.yukiko@nies.go.jp (Y.N.); hashimoto@scas.co.jp (Y.H.); 4 Department of Internal Medicine, Faculty of Medicine, Chiang Mai University, Chiang Mai 50200, Thailand; ampica.m@cmu.ac.th; 5 Department of Obstetrics and Gynecology, Faculty of Medicine, Chiang Mai University, Chiang Mai 50200, Thailand; tawiwan.p@cmu.ac.th; 6 Environmental Science Research Center, Faculty of Science, Chiang Mai University, Chiang Mai 50200, Thailand; somporn.chantara@gmail.com; 7 Faculty of Public Health, Chiang Mai University, Chiang Mai 50200, Thailand; wnaksen@gmail.com

**Keywords:** organophosphates, dialkylphosphates, neonicotinoids, insecticides, metabolites, occupational health, environmental health, reproductive hormones, steroid hormones, farmworker

## Abstract

Several studies indicated organophosphate (OP) and neonicotinoid (NEO) insecticides are endocrine disruptors; however, data are scarce. This cross-sectional study recruited 143 male farmworkers aged 18–40 years in Fang district, Chiang Mai province, northern Thailand. OP exposure was assessed by measuring urinary dialkylphosphate (DAPs) using a gas-chromatography flame photometric detector. Urinary NEOs, their metabolites (NEO/m) and serum steroid hormones were measured using liquid chromatography–tandem mass spectrometry. Characteristics of participants were determined by face-to-face interviews. DAPs and five NEO/m were detected in more than 60% of samples. The concentration of diethylphosphate was highest among DAP metabolites (geometric mean concentration (GM: 23.9 ng/mL) and the concentration of imidacloprid (IMI) was highest among NEO/m (GM: 17.4 ng/mL). Linear regression models showed that the IMI level was positively associated with testosterone, dehydrocorticosterone (DHC) and dehydroepiandrosterone (DHEA) levels. Imidacloprid-olefin and DHEA levels were positively associated. Thiamethoxam (THX) were inversely associated with DHC and deoxycorticosterone levels. Clothianidin (CLO), THX and N-desmethyl-acetamiprid levels were positively associated with the androstenedione level. CLO and THX levels were inversely associated with the cortisone level. In conclusion, the association between NEO insecticides exposure and adrenal androgens, glucocorticoids and mineralocorticoids, suggest potential steroidogenesis activities. Our findings warrant further investigation.

## 1. Introduction

Insecticides are widely used around the world in agricultural settings and households for pest control [1]. Several studies demonstrated that insecticides are linked with neurotoxicity, immunotoxicity, carcinogenesis, endocrine disruption and reproductive health effects [2,3,4,5,6,7,8]. Exposure to pesticides including organochlorine, carbamate and organophosphate (OP) is linked with alterations of hormones, including luteinizing hormone (LH), follicle-stimulating hormone (FSH), inhibin B, prolactin, estradiol and testosterone [9,10,11,12,13,14]. Several investigations have reported a decline in sperm concentrations and sperm counts related to pesticides, leading to poor semen quality and reproductive hormones alterations in males [12,15]. A recent study of farmworkers in northern Thailand investigated the associations of pesticides with testosterone [16]. It found an association between OP metabolites and total testosterone; however, statistical significance association might be random due to the small sample size [16].

A few animal studies reported that exposure to neonicotinoids (NEOs) leads to reproductive abnormalities, mostly in rats and mice. Kapoor et al. (2011) investigated the impact of oral exposure to various concentrations (5, 10 and 20 mg/kg/day) of imidacloprid (IMI) on the reproductive system of female rats and showed that rats treated with 20 mg/kg/day IMI exhibited follicular changes and reduced ovarian weight [17]. Bal et al. (2011) revealed that testosterone levels were significantly lower in male rats treated with 8 mg/kg/body weight IMI and 32 mg/kg/body weight clothianidin (CLO) than in the control group [18,19]. Bal et al. (2013) showed that the testosterone level of adult male rats orally treated with CLO did not significantly differ from that in the control group, but the reproductive system was affected [20]. Gu et al. (2013) found that exposure to IMI and acetamiprid (ACE) adversely affects fertilization in mice [21]. These findings reveal that exposure to NEOs is detrimental to mammalian sperm. In utero and lactational exposure studies have reported that low dose (1 mg/kg) and high dose (10 mg/kg) of ACE exposure in mice did not affect the testosterone levels [22]. Epidemiological studies found that maternal exposure to IMI is linked with a high risk of tetralogy of Fallot, congenital heart defects, newborn anencephaly and maternal exposure to ACE is linked with low birthweight [23,24,25,26]. A few studies demonstrated the health effects, including DNA damage and impaired lung function, of exposure to NEOs via a pesticide sprayer [27]. To the best of our knowledge, no study has investigated the associations between concentrations of NEOs and endocrine and reproductive hormones in humans.

Exposure to OP and NEO insecticides may affect steroid hormone levels. Steroids including androstenedione and dehydroepiandrosterone (DHEA) are precursors of sex hormones and are produced in the adrenal glands and gonads [28]. Although testosterone is predominantly produced in the gonads by Leydig cells, 5% is generated from the precursors androstenedione and DHEA [28]. Cortisol and cortisone are glucocorticoids and deoxycorticosterone (DOC) and dehydrocorticosterone (DHC) are mineralocorticoids released by the adrenal glands within the adrenal cortex [29,30]. However, little is known about occupational exposure to OPs and NEOs and its effects on steroid hormones.

We previously reported that Thai reproductive-aged farmworkers are exposed to OPs and NEOs based on measurement of urinary dialkylphosphates (DAPs), nonspecific metabolites of OPs, and NEOs and their metabolites (NEO/m) [31]. In that study, we found that exposure to NEOs is related to alterations of red blood cell indices in male farmworkers (a separate manuscript is being prepared). Furthermore, we did not observe any association between DAP concentrations and hematological parameters; however, previous studies reported associations between DAP metabolites and hormones [15,32,33]. Although data are available about the relationship between OP exposure and reproductive health and steroid hormones, little has been reported about the association between NEO exposure and human health. The objective of this study was to evaluate the associations between exposure to OP and NEO insecticides, assessed by measuring DAP and NEO/m concentrations and serum steroid hormones in male farmworkers.

## 2. Materials and Methods

### 2.1. Study Area and Study Participants

This cross-sectional study included reproductive-age farmworkers aged 18–40 years who worked at least 3 days per week as a farmworker and had no evidence of chronic diseases (such as diabetes, liver illness, renal insufficiency or cancer) or endocrine diseases. A total of 144 farmworkers were informed of the objectives and invited to participate in the study. Study participants were enrolled at the health-promoting hospital in Fang district, Chiang Mai province, Thailand. Participants were asked to provide written informed consent prior to face-to-face interviews using questionnaires and sample collection. One male failed to provide a blood sample, while 143 male participants provided blood samples and completed questionnaires. Each individual was directly interviewed regarding their sociodemographic characteristics and work and exposure characteristics.

### 2.2. Collection of Urine Samples

Spot urine samples were collected in 50 mL polypropylene containers. About 10 and 4 mL urine samples were required for biomonitoring of OP metabolites and NEO/m analysis, respectively. All aliquoted spot urine samples were kept at −20 °C at the field site prior to transfer to the toxicology laboratory at Research Institute for Health Sciences (RIHES), Chiang Mai University and stored at −20 °C until analyses. In addition, 4 mL urine samples were shipped on dry ice to the Health and Environmental Risk Division, National Institute Research for Environmental Studies (NIES), Tsukuba, Japan and then stored at −80 °C until analyses.

### 2.3. Collection of Blood Samples

Non-fasting blood samples were collected in the morning between 05:00 a.m. and 12:00 p.m. (four blood samples were collected after 12:00 p.m.), deposited in vacutainer tubes without anticoagulant (BD vacutainers; Becton Dickinson, NJ, USA) and centrifuged to obtain serum samples. All serum samples were kept at −20 °C at the laboratory field site prior to transfer to the toxicology laboratory at RIHES, Chiang Mai University and kept at −80 °C until analyses. In addition, all serum samples were shipped to NIES, Tsukuba, Japan and stored at −80 °C until analyses.

### 2.4. Collection of Data

The structured questionnaire was modified from the pilot study [31]. Reliability and validity were tested as quality control (QC) of the questionnaire. Each individual was directly interviewed regarding their sociodemographic factors (age, education level, monthly income), alcohol consumption, smoking status, task activities related to pesticide use (total number of years spent as a farmworker, number of days per week and hours per day worked in the field, occupational status and frequency of and last pesticide use). Trained project staff took anthropometric measurements (weight and height) and conducted the questionnaire.

### 2.5. Measurement of Urinary OP Metabolites

Urinary OP metabolites including dimethylphosphate (DMP), dimethylthiophosphate (DMTP), diethylphosphate (DEP), diethylthiophosphate (DETP) and diethyldithiophosphate (DEDTP) were measured by gas chromatography with a flame photometric detector according to the previously established method using an Agilent 7890-B GC system (Agilent Technologies, Inc., Santa Clara, CA, USA) [34]. The procedure was briefly described in our previous study [31].

### 2.6. Measurement of Urinary NEO/m

Urinary NEO/m including ACE, CLO, dinotefuran (DIN), flonicamid (FLN), IMI, nitenpyram (NIT), sulfoxaflor (SUF), thiacloprid (THI), thiamethoxam (THX), desmethyl- CLO (dm-CLO), N-desmethyl-ACE (N-dm-ACE), imidacloprid-olefin (Of-IMI) and thiacloprid-amide (THI-AM) were measured by liquid chromatography–tandem mass spectrometry (LC-MS/MS). The procedure used a Nexera liquid chromatography system coupled to a Triple Quad 8060 mass spectrometer (Shimadzu Corporations, Kyoto, Japan) and was briefly described in our previous study [31].

### 2.7. Measurement of Serum Steroid and Reproductive Hormones

Concentrations of cortisol, cortisone, DHC, testosterone, DOC, DHEA and androstenedione were measured by LC-MS/MS. The procedures were modified from previous published methods [35,36]. Briefly, 200 µL of serum was transferred to a 10 mL glass tube and 20 µL of internal standard was added. After gentle shaking, 800 µL of water:methanol:acetic acid (90:10:1, *v*/*v*/*v*) was added and mixed for 30 s. The sample was applied to a SPE cartridge, which was preconditioned with 1 mL of methanol, centrifuged at 2000× *g* for 1 min, exposed to 1 mL of 100 mM ammonium bicarbonate:25% ammonia water (50:2, *v*/*v*), centrifuged at 2000× *g* for 1 min, exposed to 1 mL of water:acetonitrile (1:9, *v*/*v*), centrifuged at 2000× *g* for 1 min and exposed to 1 mL of water:acetonitrile:acetic acid (90:10:1, *v*/*v*/*v*). The conditioned cartridge was loaded with the prepared serum sample and centrifuged at 2000× *g* for 1 min. The pretreated serum sample was washed with 1 mL of 25% methanol and centrifuged at 2000× *g* for 1 min, treated with 1 mL of 100 mM ammonium bicarbonate:25% ammonia water (50:2, *v*/*v*) and centrifuged at 2000× *g* for 1 min. Then, the pretreated serum sample and SPE cartridge were transferred to another test tube and eluted with 1 mL of water:acetonitrile (1:9, *v*/*v*). The pretreated sample was dried with a gentle nitrogen stream at 45 °C for 15 min. Residues was reconstituted with 100 µL of 30% methanol prepared with water. The eluate was injected into a Nexera liquid chromatography system coupled to a Triple Quad 8060 mass spectrometer (Shimadzu Corporations).

### 2.8. QC and Quality Assurance

#### 2.8.1. Analyses of Urinary OP Metabolites

Urine samples were pooled from anonymous non-farming volunteers and used as QC samples. QC urine samples were fortified with a standard solution containing DMP (25 ng/mL), DEP (10 ng/mL), DMTP (12 ng/mL), DMDTP (4 ng/mL), DETP (4 ng/mL) and DEDTP (2 ng/mL). These samples were analyzed for reproducibility precision. Five replicates of QC samples were analyzed by a single operator in a single day for within-day precision. For between-day precision, five QC samples were analyzed by a single operator on three consecutive days. Recovery ranged from 81.9% to 104.1%, with relative standard deviation ranging from 2.4% for DMTP to 14.2% for DEDTP for within-day precision and from 4.5% for DMTP to 20.0% for DMP for between-day precision. Six calibration points were set to range from 6.25 to 100 ng/mL for DMP, 3 to 96 ng/mL for DMTP, 1 to 32 ng/mL for DMDTP, 1.25 to 40 ng/mL for DEP, 0.25 to 64 ng/mL for DETP and 0.5 to 16 ng/mL for DEDTP with the coefficient of determination (R^2^) exceeding 0.997. The concentration of dibutyl phosphate, which was used as an internal standard, was 25 ng/mL. Method detection limits (MDLs) were defined as the concentration of a compound that yielded a signal-to-noise ratio of 3.

#### 2.8.2. Analyses of Urinary NEO/m

Urine samples were collected from pregnant volunteers from Japan and pooled as QC samples. These samples were spiked with the following concentrations of NEO/m standards: ACE, THI and SUF (0.05 ng/mL), THI-AM (0.1 ng/mL), THX (0.2 ng/mL), DIN, CLO, IMI, NIT and N-dm-ACE (0.5 ng/mL), FLN (1 ng/mL), dm-CLO (2 ng/mL) and Of-IMI (10 ng/mL). The QC samples were analyzed as part of quality quarantine and the results were recorded in a Shewhart control chart ($\bar{X}$-R control chart) based on ISO 7870 (International Organization for Standardization, Geneva, Switzerland). The MDLs were estimated using the following equation:

MDL = t_(*n*−1, 0.05)_ × 2 × s (1)

where t_(*n*−1, 0.05)_ represents the Student’s t value under an α level of 0.05 with *n* − 1 degrees of freedom and s represents the standard deviation (SD) of blank measurements in n replicates (*n*  ≥  7). Eight calibration points for each analyte were set to range from 0.0025 to 0.5 ng/mL for ACE, THI, SUF and THI-AM, 0.01 to 2.0 ng/mL for THX, 0.025 to 5.0 ng/mL for DIN, CLO, IMI, NIT and N-dm-ACE, 0.05 to 10.0 ng/mL for FLN, 0.1 to 20.0 ng/mL for dm-CLO and 0.5 to 100.0 ng/mL for Of-IMI. The concentration of the internal standard was 0.1 ng/mL for ACE and THI, 0.2 ng/mL for SUF and THI-AM, 1.0 ng/mL for DIN, CLO, IMI and NIT, 2.0 ng/mL for N-dm-ACE, 4.0 ng/mL for FLN, 8.0 ng/mL for dm-CLO and 20.0 ng/mL for Of-IMI. The R^2^ exceeded 0.99. The coefficient of variation was <14.5% for duplicate analyses of samples conducted every analytical batch of 40 samples during measurement.

#### 2.8.3. Analyses of Serum Steroid Hormones

Commercial serum samples were used as QC samples. They were spiked with the following concentrations of steroid hormone standards: 1 μg/mL for androstenedione, DHC and testosterone, 10 μg/mL for cortisol, 3 μg/mL for cortisone and DHEA and 0.3 μg/mL for DOC. The QC samples were analyzed as part of quality quarantine and mean concentrations ±10% were recorded in a control chart. All QC samples were well within the control chart during measurement day. The MDLs were estimated using Equation (1). A stock standard solution was diluted to prepare calibration curves with at least six points for each analyte with the following concentration ranges: 0.03–100 ng/mL for androstenedione and testosterone, 1–300 ng/mL for cortisol, 0.3–300 ng/mL for cortisone, 0.01–30 ng/mL for DOC, 0.1–1000 ng/mL for DHEA and 0.03–10 ng/mL for DHC, while the internal standard was used at 10 ng/mL for androstenedione and DOC, 100 ng/mL for cortisol, cortisone and DHEA and 50 ng/mL for testosterone. The R^2^ exceeded 0.99. The coefficient of variation was less than 16.3% for duplicate analysis of samples conducted every analytical batch of 50 samples during measurement.

### 2.9. Statistical Analysis

Sociodemographic characteristics of the study population were reported as the frequency distribution or mean ± SD. Correlation of general characteristics and urinary concentrations of DAPs and NEO/m were analyzed using Spearman’s correlation coefficient, with a *p*-value of less than 0.05 considered significant. Urinary concentrations of DAPs and NEO/m were normalized relative to specific gravity (SG) in the same samples to adjust for urine dilution; these concentrations were therefore reported as ng/mL. Concentrations were corrected for SG using the following adapted formula [37]:

P_c_ = P[(SG_Med_ − 1/SG_Meas_ − 1)] (2)

where P_c_ is the SG-corrected metabolite concentration, P is the observed metabolite concentration, SG_Med_ is the median SG of all samples tested in the study and SG_Meas_ is the measured SG of the individual urine sample. The concentrations of DAPs and NEO/m were assessed to check normality using the Kolmogorov–Smirnov test and log10-transformed to obtain normal distributions before statistical analyses. Summary statistics were computed using the NADA package (version 1.6-1.1) in the statistical R software. Geometric means (GM) and geometric standard deviations (GSDs) were calculated for analytes detected in >50% of samples. The total concentrations of dimethylalkylphosphates (sumDMP), diethylalkylphosphates (sumDEP) and DAPs (sumDAP) were used to determine the association between OP exposure and steroid hormones. To calculate total concentrations of sumDMP, sumDEP and sumDAP, mass concentrations in urine (ng/mL) were converted to molar concentrations (nmol/L) with correction for SG using the following molecular masses in g/mol: 126.05 (DMP), 142.10 (DMTP), 158.18 (DMDTP), 154.10 (DEP), 169.16 (DETP) and 186.23 (DEDTP). SumDMP was defined as the molar sum of DMP, DMTP and DMDTP. SumDEP were defined as the molar sum of DEP, DETP and DEDTP. SumDAP was defined as the molar sum of all DAP metabolites. The concentrations of sumDMP, sumDEP and sumDAP in nmol/L and the concentrations of CLO, IMI, THX, N-dm-ACE and Of-IMI in ng/mL were treated as continuous variables (log10-transformed) in subsequent analysis. The concentrations of androstenedione, cortisol, cortisone, DHEA, DHC, DOC and testosterone were also log-transformed to reduce skewness. Subject-related sociodemographic characteristics and work, exposure and reproductive health characteristics of the study population with missing data were computed by the multivariate imputation by chained equations (MICE) method, with 10 imputations and 10 iterations using MICE package (version 3.13.0) prior to multivariate linear regression analysis. In addition, detected urinary concentrations below the MDL were imputed with quantile regression approach for left-censored missing (QRILC) for subsequent statistical analysis. Linear regression models were used to assess the association between each DAP and NEO/m concentrations and steroid hormone concentrations, with the α level set to 0.05. In each model, the urinary DAP and NEO/m concentration was the independent variable while the serum steroid hormone was the dependent variable. Each model was adjusted for age, body mass index (BMI), smoking status, alcohol consumption, ethnicity, education level, monthly income, total number of years spent as farmworker, status of farmworker, number of days per week and hours per day worked in the field, duration of last pesticide use prior to sample collection and hematological status based on current scientific knowledge [9,16].

## 3. Results

### 3.1. Characteristics of Study Participants

The general characteristics of the study population are provided in Table 1. This reproductive-age population had a mean (SD) age of 30.1 (5.8) years. About 50% of participants were normal weight, while some participants were obese class I (19%) and overweight (18%) according to the Regional Office for the Western Pacific standard [38]. In total, 56% of participants had no formal education, 28% completed primary school and less than 9% completed high school, had a technical/professional education or attained a university or higher degree. The monthly individual income of participants was ~300 USD (9200 THB). In total, 54% of participants were current smokers, 13% were former smokers, 33% were nonsmokers and 85% consumed alcohol. The Spearman’s correlation coefficients between urinary DAP and NEO/m and general characteristics are provided in Table S1. Correlation was negligible between BMI and urinary concentrations of sumDMP, sumDEP, sumDAP, THX, N-dm-ACE and Of-IMI (correlation coefficient = −0.19, −0.27, −0.27, −0.09, −0.26, −0.23, respectively). Negligible correlation was also found in the relationship between monthly income and urinary concentrations of sumDEP, sumDAP, IMI and Of-IMI (correlation coefficient = 0.14, 0.15, 0.11, 0.15, respectively). There was a negligible correlation between smoking status and urinary concentration of sumDMP, sumDAP, and Of-IMI (correlation coefficient = 0.19, 0.17, 0.17, respectively). No correlations were observed between DAP and NEO/m and alcohol consumption. There was a weak negative correlation between educational level and urinary concentration of sumDMP, sumDEP, sumDAP, IMI and Of-IMI (correlation coefficient = −0.32, −0.32, −0.35, −0.24, −0.30, respectively). In the study area, OP, mainly chlorpyrifos, dichlorvos, ethion, profenofos, triazophos, and NEO insecticides including IMI and DIN are commonly used which is evidenced by a local retailer shop in the community.

### 3.2. Urinary DAP and NEO/m Concentrations

Table 2 shows the detection frequencies and the GMs, selected percentiles and maximums of urinary DAP and NEO/m concentrations. DAP metabolites with diethyl moieties were mostly detected at higher concentrations than those with dimethyl moieties. Five NEO/m (CLO, IMI, THX, N-dm-ACE and Of-IMI) were detected at higher than the MDL in more than 60% of cases and were analyzed further. The GM concentration of DETP was highest (23.9 ng/mL), followed by DEP (20.7 ng/mL) and DEDTP (9.3 ng/mL). Among NEO/m, the GM concentration of IMI was highest (17.4 ng/mL), followed by N-dm-ACE (15.8 ng/mL), THX (9.1 ng/mL), CLO (7.4 ng/mL) and Of-IMI (5.1 ng/mL).

### 3.3. Serum Steroid Hormone Concentrations

The distributions of all measured steroid and reproductive hormone levels in farmworkers are provided in Table 3. The detection rates of androstenedione, cortisol, cortisone, DHC, DHEA, DOC and testosterone were 100% among 143 farmworkers. The mean concentrations of androstenedione, cortisol, cortisone, DHC, DHEA, DOC and testosterone were 0.8, 140, 24.7, 1.0, 3.3, 0.9 and 6.5 ng/mL, respectively.

### 3.4. Associations between DAP and NEO/m Concentrations and Serum Steroid Hormones 

**Figure 1:**
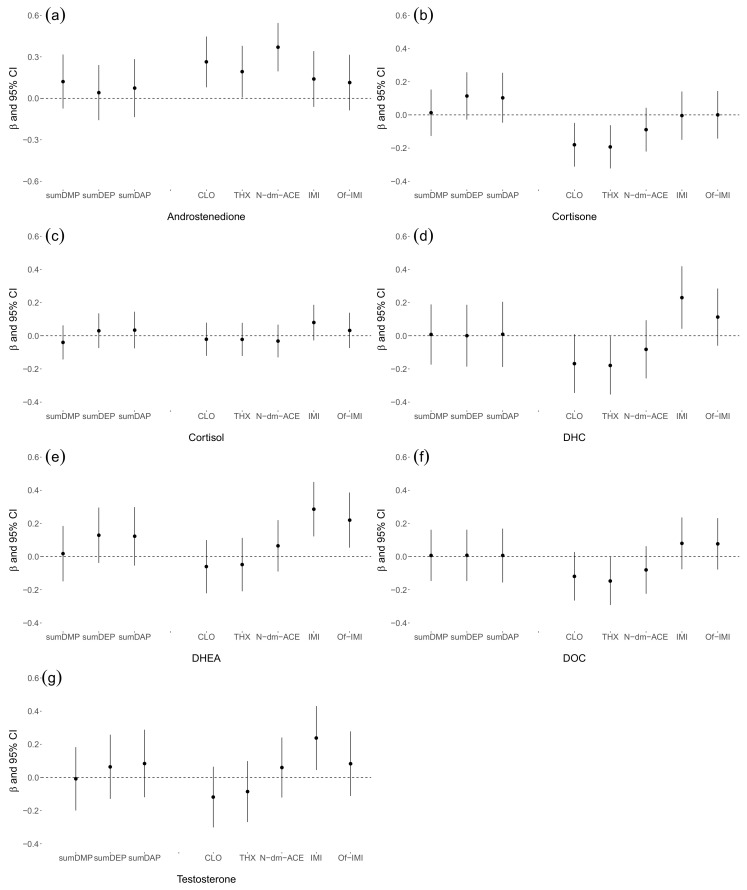
Linear regression models analyzing the association between each urinary concentration of DAP and NEO/m and serum levels of androstenedione (**a**), cortisol (**b**), cortisone (**c**), dehydrocorticosterone (**d**), dehydroepiandrosterone (**e**), deoxycorticosterone (**f**) and testosterone (**g**). The standardized partial regression coefficient (β) and 95% confidence interval (CI) were presented as average change in dependent variable per one-unit change in the independent variable. The urinary concentrations of DAP were grouped as total of three dimethylalkylphosphate (sumDMP: DMP+DMTP+DMDTP), total of three diethylalkylphosphate (sumDEP: DEP+DETP+DEDTP), total of dialkylphosphates (sumDAP: sumDMP+sumDEP) and the urinary concentrations of NEO/m were shown as individual including CLO, THX, N-dm-ACE, IMI and Of-IMI. The same model was fitted for all the exposure measures: each urinary concentration of DAP and NEO/m was introduced into the linear regression model separately and adjusted for age, body mass index, smoking status, alcohol consumption, ethnicity, education level, monthly income, total number of years spent as a farmworker, status of farmworker, number of days per week and hours per day worked in the field, duration of last pesticide used prior to sample collection and hematological status. The independent variables were log10-transformed urinary concentrations of DAPs and NEO/m and the dependent variables were log-transformed serum steroid hormone. Abbreviations: DAP, dialkylphosphate; sumDMP, total of dimethylalkylphosphates; sumDMP, total of diethylalkylphosphates; sumDAP, total of dialkylphosphates; NEO/m, neonicotinoids and their metabolites; CLO, clothianidin; IMI, imidacloprid; THX, thiamethoxam; N-dm-ACE, N-desmethyl-acetamiprid; Of-IMI, imidacloprid-olefin; β, standardized partial regression coefficient; CI, confidence interval.

Linear regression models for each urinary DAP and NEO/m concentrations and association with serum steroid hormones are shown in Figure 1. The urinary concentrations of DAP were grouped as total of three dimethylalkylphosphate (sumDMP: DMP+DMTP+DMDTP), total of three diethylalkylphosphate (sumDEP: DEP+DETP+DEDTP), total of dialkylphosphate (sumDAP: sumDMP+sumDEP) and the urinary concentrations of NEO/m were shown as individual including CLO, THX, N-dm-ACE, IMI and Of-IMI. The same model was fitted for all the exposure measures: each urinary DAP and NEO/m concentration was introduced into the linear regression model separately and adjusted for age, body mass index, smoking status, alcohol consumption, ethnicity, education level, individual income, total number of years spent as a farmworker, occupational status, number of days per week and hours per day worked in the field, duration of last pesticide use prior to sample collection and hematological status. In each model, log10-transformed urinary concentration of DAPs and NEO/m was the independent variable whereas log-transformed serum steroid hormone was the dependent variable. Androstenedione levels showed a significant positive association with CLO, THX and N-dm-ACE concentrations. Cortisone levels showed a significant negative association with CLO and THX concentrations. DHEA levels showed a significant positive association IMI and Of-IMI concentrations. DHC and testosterone levels were positively associated with IMI concentrations. DHC and DOC levels were negatively associated with THX concentration. No significant associations were observed between concentrations of steroid hormones and DAPs.

## 4. Discussion

Linear regression models revealed that NEO/m concentrations were associated with serum steroid hormone concentrations. NEOs are classified as systemic insecticides [39] that are widely spread throughout the environment, which may explain why significant associations were found for concentrations of NEO/m but not for concentrations of OP metabolites. Among NEO/m, the IMI level was positively associated with the DHC, DHEA and testosterone levels and there was a positive association between Of-IMI and DHEA levels. The THX level was negatively associated with the DHC and DOC levels. There were positive associations between the CLO, THX and N-dm-ACE levels and the androstenedione level as well as a negative association between the CLO and THX levels and the cortisone level. To the best of our knowledge, this is the first study to evaluate the effects of NEO/m concentrations on human serum steroid hormones. Our findings suggest that NEO exposure is associated with disruption of endocrine and reproductive functions.

### 4.1. Serum Steroid Hormones

A recent study reported that the normal reference range of the serum testosterone level is 2.1–9.7 ng/mL in fertile men (*n* = 120) [40]. In our study, the mean testosterone level was 6.5 ng/mL among male farmworkers. This is slightly higher than the mean level in the previous report (4.8 ng/mL). Most participants in the previous study were overweight or obese and a negative correlation between BMI and the testosterone level has been reported [40,41]. Furthermore, an epidemiological study reported that the mean testosterone levels in Thai male farmers and Venezuelan farmworkers were 7.4 and 6.7 ng/mL, respectively, which is similar to the level in our study [12,16]. In comparison with our study, a lower testosterone level (4.5 ng/mL) was reported in Mexican male floriculture workers during intense pesticide spraying, but a similar level (7.6 ng/mL) was reported during less intense pesticide spraying [9]. This indicates that testosterone levels in our participants were within the normal range according to the World Health Organization guidelines and higher levels were found in males who had worked in agricultural fields for at least 1–20 years [9,16,42].

In addition to testosterone, the present study is the first to report the concentrations of several steroid hormones including androstenedione, cortisol, cortisone, DHC, DHEA and DOC among male farmworkers. Their concentrations were within the reference intervals compared with previously published values [28,43].

### 4.2. Associations between OP and NEO Exposure and Adrenal Androgen Hormones

No epidemiological study has demonstrated an association between exposure to NEO insecticides and steroid hormones [44]. Most studies of reproductive toxicity induced by NEO insecticide used animals and focused on IMI and CLO. To the best of our knowledge, this is the first study to report associations between urinary NEO/m concentrations and adrenal androgen hormones including androstenedione, DHEA and testosterone in humans. We found positive associations of IMI and Of-IMI with DHEA (β = 0.29; 95% CI: 0.12, 0.45, β = 0.22; 95% CI: 0.05, 0.39, respectively), and exposure to IMI and Of-IMI tended to be associated with an increasing androstenedione concentration. The testosterone level was positively associated with IMI and Of-IMI concentrations (β = 0.24; 95% CI: 0.04, 0.43, β = 0.08; 95% CI: −0.11, 0.28, respectively), which is inconsistent with animal studies [18]. A previous experimental study of male Wistar rats exposed to 45 and 90 mg/kg/body weight IMI reported decreased enzymatic activity of 3β-hydroxysteroid dehydrogenase (3β-HSD) and 17β-hydroxysteroid dehydrogenase (17β-HSD), which catalyze conversion of DHEA to androstenedione and of androstenedione to testosterone, respectively, resulting in decreased testosterone concentrations [28,45]. The increased concentrations of DHEA, androstenedione and testosterone associated with exposure to IMI and Of-IMI suggest that adrenal androgen synthesis is promoted via induced expression of 3β-HSD type 2 (HSD3B2) and high CYP17A activity [46]. CYP17 enzymes including 17α-hydroxylase and 17,20-lyase catalyze a step involved in formation of DHEA.

There were positive associations between CLO, THX and N-dm-ACE levels and the androstenedione level (β = 0.26; 95% CI: 0.08, 0.45, β = 0.19; 95% CI: 0.01, 0.38, β = 0.37; 95% CI: 0.20, 0.54, respectively). However, exposure to CLO, THX and N-dm-ACE tended to be associated with decreasing DHEA and testosterone concentrations. HSD3B2 influences synthesis of DHEA (adrenal androgen), aldosterone (mineralocorticoid) and cortisol (glucocorticoid) by competing with CYP17. Additionally, low HSD3B2 expression and high CYP17 activity facilitates production of androgen, while high HSD3B2 expression and low CYP17 activity facilitates production of aldosterone [46]. CLO, THX and N-dm-ACE likely induce aldosterone production due to low CYP17 activity, which decreases the DHEA level, elevates HSD3B2 expression and consequently increases the androstenedione level. Interestingly, both CYP17 activity and HSD3B2 play an important role in regulation of adrenal androgen production and different NEO insecticides influenced CYP17 activity disparately in our study.

17β-HSD is involved in formation of testosterone and has five isoforms [47]. A previous study of human tissues showed that 17β-HSD type 2 (HSD17B2) degrades estradiol into estrone (estrogens) and testosterone into androstenedione. In addition, 17β-HSD type 3 (HSD17B3) and type 5 (HSD17B5) catalyze formation of testosterone in testes and peripheral tissues, respectively [47]. We speculate that exposure to different NEOs interferes with 17β-HSD gene expression in a different manner, resulting in different effects on the testosterone level. Moreover, the testosterone level could be altered due to changes in the LH level upon exposure to insecticides [9]. Our findings suggest that human exposure to NEOs induces the hypothalamic–pituitary–adrenal axis through adrenal androgen biosynthesis pathways. Further studies are needed to confirm our findings and to elucidate the underlying mechanisms.

Another possible explanation for association between exposure to NEO and adrenal androgen hormones is NEO agonistic or antagonistic interaction with hormone receptors including androgen receptor (AR) and estrogen receptor (ER) [48]. Both AR and ER are potentially involved in mediating the action of sex steroid hormones [49,50]. An animal study on male mice showed IMI decreases testosterone, can interact with AR and decrease AR expression in the testes [51]. A study of endocrine activities of THX and ACE found in waste water effluent using yeast-based bioassays showed weak antagonist androgen activity and antagonist estrogen activity for THI [52]. The molecular study of Zhang et al. (2020) revealed that IMI and THI can interfere with estrogen induced signaling [48]. Therefore, NEO mimics or disturbs the action of natural hormones, which may alter the synthesis, metabolism, transport of the hormones. We speculate that NEO may have agonist or antagonist activities at the AR and ER which caused alteration on the steroid and reproductive hormones syntheses, metabolisms and transport of the hormones. Due to limited information, more research is needed to elucidate the endocrine activity of NEO insecticides.

Several previous epidemiological studies found associations between testosterone and exposure to OP insecticides by measuring DAP metabolites in urine [9,15,16,53]. In our study, there was no significant association between testosterone level and urinary DAP concentrations. These findings are consistent with a previous study of Mexican agricultural workers, which suggested that OP metabolites overall have lesser effects than specific metabolites [53]. Furthermore, significant positive associations between concentrations of OP metabolites and testosterone were observed among Thai farmers and Mexican floriculture workers [14,16]. However, a longitudinal study showed a significant negative association between concentrations of OP metabolites and the testosterone level [9]. A decreased testosterone level associated with the chlorpyrifos concentration was observed among fertile and infertile men [10].

### 4.3. Associations between OP and NEO Exposure and Glucocorticoids and Mineralocorticoids 

In this study, CLO and THX concentrations were inversely associated with the cortisone concentration, inconsistent with an animal study [54]. In that study, treatment of rats with IMI resulted in stress and increased the cortisone level, suggesting that IMI disrupts glucose homeostasis [54]. These findings indicate that exposure to NEO insecticides alters glucocorticoids including cortisone and mineralocorticoids including DHC and DOC. Furthermore, we did not find any association between DAPs and steroid hormones. Further research is warranted to reveal the endocrine disrupting activities of OPs and NEOs among the general population.

### 4.4. Limitations

This study found associations between NEO exposure and steroid hormones including androstenedione, DHEA, DHC, DOC, cortisone and testosterone, but has some limitations. First, this cross-sectional study limited causal inference. Furthermore, prospective studies are needed to confirm the results. DAPs and NEOs have short half-lives in humans [55,56]. Data regarding historic pesticide use must be collected. Previous studies found that urinary concentrations of DAP metabolites and some NEO/m do not adequately reflect exposure for over 1 month [57,58]. Spot urine and blood samples were collected after breakfast and before lunch to keep the sampling period short. OP and NEO insecticides have a toxic effect on the hypothalamic pituitary gonadal (HPG) axis, which regulates the synthesis and secretion of sex hormones [9]. The HPG axis-involved hormones including gonadotropin releasing hormones, LH, FSH, inhibin B, progesterone and estradiol should be investigated to clarify the mechanism of OP and NEO impacts on these hormones. Another limitation is that the adjusted R^2^ was relatively small in multivariate regression models, particularly for androstenedione and testosterone. A further study should consider potential factors affecting the androgen axis.

## 5. Conclusions

The key result of this study is that exposure to NEO insecticides is associated with steroid hormones among male farmworkers. IMI and Of-IMI levels were positively associated with androstenedione, cortisol, cortisone, DHEA, DHC, DOC and testosterone levels; however, the levels of CLO, THX and N-dm-ACE showed a negative trend with levels of hormones, except for androstenedione. We speculate that exposure to different NEO insecticides elicits opposite effects on expression of steroid enzymes and hormone receptor binding. Exposure to systemic insecticides disrupts steroidogenesis and may adversely affect the reproductive system among people of reproductive age. There are no previous human studies of this topic, and the results of animal studies are inconsistent with our human study. Therefore, human studies of exposure to OP and NEO insecticides and its impact on steroid hormones are needed in other populations.

## Figures and Tables

**Table 1:** General characteristics of the study populations (*n* = 143).

General Characteristics	*n* (%)
Age (18–40 years) (*n* = 143)	
Mean (SD)	30.1 (5.8)
Body mass index (kg/m^2^) (*n* = 142)	
Mean (SD)	23.5 (4.3)
<18.5 (underweight)	5 (3.5)
18.5–23.0 (normal weight)	71 (50.0)
23.0–25.0 (overweight)	26 (18.2)
25.0–30.0 (obese class I)	27 (18.9)
≥30.0 (obese class II)	12 (8.4)
NA	2 (1.4)
Ethnicity (*n* = 143)	
Thai	11 (7.7)
Other (Hmong, Tai Yai, Burmese, Palong, Lahu)	132 (92.3)
Educational level (*n* = 143)	
Illiterate/no formal education	81 (56.6)
Primary school	40 (28.0)
High school	12 (8.4)
Technical/professional	5 (3.5)
University or higher degree	5 (3.5)
Individual income (*n* = 139)	
Mean (SD) (THB/month)	9200 (12,000)
Smoking status (*n* = 143)	
Never smoked/nonsmoker	48 (33.6)
Former smoker	18 (12.6)
Current smoker	77 (53.8)
Alcohol consumption (*n* = 143)	
No	22 (15.4)
Yes	121 (84.6)
Sampling characteristics	
Month of sampling (*n* = 143)	
June	27 (16.8)
July	84 (58.7)
August	29 (20.3)
September	6 (4.2)
Time of urine collection (*n* = 143)	
05:00–12:00	137 (95.8)
12:01–18:00	6 (4.2)
Time of blood collection (*n* = 143)	
05:00–12:00	139 (97.2)
12:01–18:00	4 (2.8)
Total number of years spent as a farmworker	
Mean (SD)	8.9 (6.4)
Total number of days per week worked in the field	
Mean (SD)	8.1 (1.8)
Total number of hours per day worked in the field	
Mean (SD)	5.5 (1.7)
Status of farmworker	
Working on own farm or family farm; owner	64 (45.0)
Working on another person’s farm; permanent laborer	63 (44.4)
Working on a rented farm	15 (10.6)
NA	1 (0.7)
Last pesticide use	
Three days prior to urine collection and the day of urine collection	66 (46.2)
Two weeks ago	24 (16.8)
One month ago	5 (3.5)
>One month ago	14 (9.8)
Do not know	34 (23.8)

Abbreviations: SD, standard deviation; USD, United States Dollar; THB, Thai Baht; NA, no answer. Results are reported as mean (SD) or *n* (%).

**Table 2:** Detection frequency and geometric mean (GM), geometric standard deviation (GSD), minimum, maximum and selected percentiles of urinary DAP and NEO/m concentrations among reproductive-age farmworkers (*n* = 143).

Compound (ng/mL)	MDL	>MDL (%)	GM	GSD	Quartiles				
					P25	P50	P75	P95	Max.
DAP:									
DMP	5.0	28.7	-	-	-	-	-	12.6	73.4
DMTP	1.0	44.8	-	-	-	-	2.8	37.0	134
DMDTP	0.5	25.2	-	-	-	-	-	5.2	398
DEP	1.0	100.0	20.7	4.5	3.8	9.5	24.7	150	5678
DETP	0.125	99.3	23.9	4.2	3.1	7.4	25.3	123	445
DEDTP	0.25	79.0	9.3	6.1	0.7	2.2	11.9	67.3	386
NEO/m:									
ACE	0.0011	46.9	-	-	-	-	0.006	0.1	1.5
CLO	0.007	96.5	7.4	3.6	0.04	0.1	0.2	1.0	14.6
DIN	0.003	16.1	-	-	-	-	-	0.3	1.0
FLN	0.01	16.8	-	-	-	-	-	-	0.02
IMI	0.009	99.3	17.4	5.5	0.06	0.2	0.9	4.7	24.6
NIT	0.007	3.5	-	-	-	-	-	-	0.3
THI	0.0009	4.9	-	-	-	-	-	-	0.009
THX	0.003	97.2	9.1	4.9	0.03	0.07	0.3	1.9	75.8
SUF	0.002	35.7	-	-	-	-	-	0.04	0.3
dm-CLO	0.03	29.4	-	-	-	-	0.07	0.6	4.1
N-dm-ACE	0.007	99.3	15.8	3.7	0.1	0.3	0.6	4.1	18.1
Of-IMI	0.2	64.3	5.1	5.3	-	0.7	3.5	17.9	253
THI-AM	0.002	0.7	-	-	-	-	-	0.02	0.04

Abbreviations: MDL, method detection limit; ng/mL, nanograms per milliliter; GM, geometric mean; GSD, geometric standard deviation; P25, 25th percentile; P50, 50th percentile; P75, 75th percentile; P95, 95th percentile; Max., maximum concentration; DAP, dialkylphosphate; DMP, dimethylphosphate; DMTP, dimethylthiophosphate; DMTDP, dimethyldithiophosphate; DEP, diethylphosphate; DETP, diethylthiophosphate; DETDP, diethyldithiophosphate; NEO/m, neonicotinoids and their metabolites; ACE, acetamiprid; CLO, clothianidin; DIN, dinotefuran; IMI, imidacloprid; NIT, nitenpyram; SUF, sulfoxaflor; THI, thiacloprid; THX, thiamethoxam; FLN, flonicamid; N-dm-ACE, N-desmethyl-ACE; dm-CLO, desmethyl-clothianidin; THI-AM, thiacloprid-amide; Of-IMI, imidacloprid-olefin; -, values lower than MDLs.

**Table 3:** Steroid hormone concentrations in the study population (*n* = 143).

Steroid Hormone (ng/mL)	Mean (SD)	Min.	P25	P50	P75	P95	Max.
Androstenedione	0.8 (0.3)	0.3	0.6	0.7	1.0	1.4	2.4
Cortisol	140 (67.6)	5.5	91.9	127	178	278	379
Cortisone	24.7 (9.1)	1.0	18.5	23.0	29.8	42.6	55.1
DHC	1.0 (0.7)	0.07	0.5	0.8	1.3	2.2	3.4
DHEA	3.3 (1.8)	0.5	2.0	2.9	4.4	6.7	10.0
DOC	0.9 (0.5)	0.1	0.5	0.8	1.1	1.7	3.6
Testosterone	6.5 (3.1)	1.5	4.4	5.9	7.9	12.1	19.9

Abbreviations: SD, standard deviation; ng/mL, nanograms per milliliter; Min, minimum concentration; P25, 25th percentile; P50, 50th percentile; P75, 75th percentile; P95, 95th percentile; Max. maximum concentration; DHC, dehydrocorticosterone; DHEA, dehydroepiandrosterone; DOC, deoxycorticosterone.

## Supplementary Materials

The following are available online at https://www.mdpi.com/article/10.3390/ijerph18115599/s1, Table S1: Spearman’s correlation coefficients between general characteristics and urinary DAP and NEO/m concentrations.
