# Supplementary material for: Exposure to Organophosphate and Neonicotinoid Insecticides and Its Association with Steroid Hormones among Male Reproductive-Age Farmworkers in Northern Thailand
Source: Int J Environ Res Public Health. 2021 May 24;18(11):5599. doi: 10.3390/ijerph18115599 (PMC8197278; doi:10.3390/ijerph18115599)
Supplement: Supplementary file 1 [file ijerph-18-05599-s001.zip › ijerph-1192712-supplementary.pdf]

Supplementary materials

Exposure to Organophosphate and Neonicotinoid Insecticides and its Association with Steroid Hormones among Thai Male Reproductive-Age Farmworkers

Neeranuch Suwannarin [1,2,3], Tippawan Prapamontol [2,*], Tomohiko Isobe [3], Yukiko Nishihama [3], Yuki Hashimoto [3], Ampica Mangklabruks [4], Tawiwan Pantasri [5], Somporn Chantara [6], Warangkana Naksen [7], Shoji F. Nakayama [3,*]

[1] PhD Degree Program in Environmental Science, Environmental Science Research Center, Faculty of Science, Chiang Mai University, Chiang Mai 50200, Thailand; suwannarin.ns@gmail.com

[2] Research Institute for Health Sciences (RIHES), Chiang Mai University, Chiang Mai 50200, Thailand

[3] Health and Environmental Risk Division,, National Institute for Environmental Studies, Tsukuba, Ibaraki 305-8506, Japan; isobe.tomohiko@nies.go.jp (T.I.); nishihama.yukiko@nies.go.jp (Y.N.); hashimoto@scas.go.jp (Y.H.)

[4] Department of Internal Medicine, Faculty of Medicine, Chiang Mai University, Chiang Mai 50200, Thailand; ampica.m@cmu.ac.th (A.M.)

[5] Department of Obstetrics and Gynecology, Faculty of Medicine, Chiang Mai University, Chiang Mai 50200, Thailand; tawiwan.p@cmu.ac.th (T.P.)

[6] Environmental Science Research Center, Faculty of Science, Chiang Mai University, Chiang Mai, 50200, Thailand; somporn.chantara@gmail.com (S.C.)

[7] Faculty of Public Health, Chiang Mai University, Chiang Mai 50200, Thailand; wnaksen@gmail.com (W.N.)

* Correspondence: tippawan.prapamontol@cmu.ac.th (T.P.); fabre@nies.go.jp (S.F.N.); Tel.: +81-29-850-2786 (S.F.N.)

Table S1 Spearman's correlation coefficients between general characteristics and urinary DAP and NEO/m concentrations.

Parameters	BMI		Education		Monthly income		Smoking status		Alcohol consumption	
	**r**	**p-value**	**r**	**p-value**	**r**	**p-value**	**r**	**p-value**	**r**	**p-value**
**DAP:**										
sumDMP	-0.192	0.022	-0.315	<0.001	0.111	0.061	0.192	0.022	0.009	0.916
sumDEP	-0.273	0.001	-0.317	<0.001	0.143	0.015	0.135	0.108	-0.154	0.067
sumDAP	-0.267	0.001	-0.354	<0.001	0.154	0.009	0.169	0.043	-0.157	0.062
**NEO/m:**										
CLO	-0.055	0.510	-0.079	0.351	0.072	0.220	0.084	0.317	-0.017	0.843
THX	-0.093	<0.001	-0.067	0.428	0.032	0.590	0.141	0.093	-0.015	0.858
IMI	-0.297	0.270	-0.242	0.004	0.117	0.048	0.111	0.188	-0.158	0.059
N-dm-ACE	-0.257	0.002	-0.315	<0.001	0.111	0.061	0.130	0.121	-0.153	0.068
Of-IMI	-0.234	0.005	-0.317	<0.001	0.143	0.015	0.173	0.038	-0.105	0.210

Abbreviations: DAP, dialkylphosphate; sumDMP, total of dimethylalkylphosphates; sumDMP, total of diethylalkylphosphates; sumDAP, total of dialkylphosphates; NEO/m, neonicotinoids and their metabolites; CLO, clothianidin; IMI, imidacloprid; THX, thiamethoxam; N-dm-ACE, N-desmethyl-acetamiprid; Of-IMI, imidacloprid-olefin; BMI, Body Mass Index; r, correlation coefficient.
